# Lower cyclooxygenase-2 expression is associated with recurrence of solitary non-muscle invasive bladder carcinoma

**DOI:** 10.1186/1746-1596-7-152

**Published:** 2012-11-05

**Authors:** Tomislav Tadin, Kristian Krpina, Sanja Štifter, Emina Babarović, Željko Fučkar, Nives Jonjić

**Affiliations:** 1 Ultrasound Diagnostic Service, Health Centre Rijeka, Martina Kontuša 18, Rijeka, 51000, Croatia; 2 Department of Urology, University Hospital Centre Rijeka, Tome Strižića 3, Rijeka, 51000, Croatia; 3 Department of Pathology, Faculty of Medicine, University of Rijeka, B. Branchetta 20, Rijeka, 51000, Croatia; 4 Tomislav Tadin, MD M.Sc., Ultrasound Diagnostic Service, Martina Kontuša 18, Rijeka, 51000, Croatia

**Keywords:** Non-muscle invasive bladder cancer, Recurrence, Cyclooxygenase-2, Tumor infiltrating lymphocytes

## Abstract

**Background:**

A new modality is necessary to prevent recurrence of superficial bladder cancer after complete transurethral resection because of the high recurrence rate even with current prophylaxis protocols.

**Methods:**

In order to analyze the predictive value of cyclooxygenase-2 (COX-2) expression and tumor infiltrating lymphocytes (TILs) in recurrence of this disease tumor specimens from 127 patients with solitary papillary non-muscle invasive bladder cancer (NMIBC), 78 with recurrent disease and 49 without recurrence during follow up of minimum 5 years, were retrieved for tissue microarrays construction and immunohistochemical analysis. COX-2 expression was scored according to Allred’s scoring protocol, while presence of TILs was categorized as absent (no) or present (yes) on whole tissue sections.

**Results:**

COX-2 immunoreactivity was presented in 70 (71%), weak in 16% and strong in 55% of cases, while 29 (29%) tumors were negative. TILs were present in 64 (58%) NMIBC, while 44 cases (41%) did not reveal mononuclear infiltration in tumoral stroma. Statistical analysis demonstrated a higher proportion of patients with recurrence in the group with the COX-2 score 0, and lower in the group with score 2 (p=0.0001, p=0.0101, respectively). In addition, a higher proportion of recurrent patients in the group with no TILs, and lower proportion in the group with TILs were found (p=0.009, p=0.009, respectively). Univariate and multivariate analysis revealed overexpression of COX-2 and presence of TILs as negative predictors.

**Conclusion:**

Patients with lower COX-2 expression and absence of TILs in NMIBC need to be followed up more vigorously and probably selected for adjuvant therapy.

**Virtual slide:**

The virtual slide(s) for this article can be found here: http://www.diagnosticpathology.diagnomx.eu/vs/1411318819790406

## Background

Urinary bladder cancer (UBC), on the average, includes 2% of all the malignant diseases with male-to-female ratio being about 4:1. The incidence of UBC increases with age [1]. The mortality from transitional cell carcinoma (TCC) of the urinary bladder increases significantly with the progression of superficial to invasive disease. Approximately 75-85% of patients present with a non-muscle invasive bladder carcinoma (stages pTa, pT1, pTis). Despite the same category this is a very heterogeneous group of tumors with various biological outcomes. The main clinical feature of UBC is a high percentage of recurrence [2]. Carcinoma of the urinary bladder is the only malignant neoplasm for which immunotherapy is often included as part of standard care. Intravesical instillations of Bacille Calmette–Guerin (BCG) has been demonstrated to reduce the recurrence rate and the risk of progression to muscle-invasive disease in patients with carcinoma in situ (pTis), as well as non-muscle-invasive urothelial carcinomas [3,4]. BCG immunotherapy results in 70% to 75% and 50% to 60% complete response rates for carcinoma in situ and small residual tumors, respectively [5]. Unfortunately, a significant percentage of patients will fail initial BCG therapy. These patients would have much more benefit if they were oriented early to other therapeutic approaches. In addition, another 30% to 50% of BCG responders will develop recurrent tumors within 5 years [6,7].

In fact, 70% of patients treated with transurethral resection (TUR) experience a relapse of the underlying disease and 15-25% of patients will progress over time to muscle-invasive cancer [2]. Although some prognostic variables have been shown to predict recurrence and can be used to identify patients who require adjuvant therapy after TUR, additional reliable markers for disease progression and recurrence are needed [8-10].

Standard prognostic factors like histological grading are limited in predicting possible recurrence of the disease. So, understanding of molecular processes that could reflect individual biological potential and clinical behavior is important. For that purpose, various biomarkers have been investigated since they have potential in decoding unique biological features in identifying patients with high risk of progression after the local treatment, as well as in more reliable prognosis and treatment of UBC [11]. In the present study the cyclooxygenase-2 (COX-2) was investigated. Beside prostaglandin G/H synthases and COX-1 it is one of the key enzymes in the synthesis of prostaglandins from arachidonic acid. COX-2 is not expressed in most tissue under normal conditions, but expression is rapidly induced by growth factors or agents that cause tissue irritation or inflammation [12]. COX-2 is expressed in many solid as well as hematological malignancies [13] where prostaglandins have been reported to increase proliferation, enhance angiogenesis, promote invasion, and inhibit apoptosis and differentiation, thus participating in carcinogenesis through various mechanisms [13,14]. There is evidence that COX-2 expression and activity is also important in the development of UBC [15,16].

To determine if COX-2 overexpression could be causally related to recurrence of superficial UBC the present study was designed with pathologically homogenous group of solitary papillary non-muscle invasive bladder cancer (NMIBC) presented in patients according to development of the recurrent disease. More specifically, the aim was to assess and compare the COX-2 expression between the patients with and without recurrence of disease and, moreover, to analyze association between COX-2 expression and tumor infiltrating lymphocytes (TILs). We hypothesize that such investigation can be used to predict recurrence and help identify patients who require adjuvant treatment.

## Materials and methods

### Clinicopathological data

The tumor specimens analyzed in this study were obtained from a total of 127 patients with solitary papillary NMIBC treated with initial transurethral resection (TUR), as a standard procedure at the Department of Urology, Rijeka University Hospital Center in Rijeka, between 1996 and 2006. None of the patients received adjuvant chemotherapy or immunotherapy or any other medical intervention after the initial TUR. All patients with multiple tumors and patients with a solid or flat aspect of the tumor were excluded from this study. The study was approved by the University of Rijeka Ethics Committee.

All the sections were reviewed to confirm the original diagnosis and were staged according to the 2002 American Joint Committee on Cancer guidelines [17] and graded according to the 2004 World Health Organization classification system [18] by an expert urologic pathologist. All the tumors were classified as papillary urothelial neoplasm of low malignant potential (PUNLMP) or low-grade papillary urothelial carcinoma (LGPUC). Clinicopathologic data were obtained from patient medical records and from the files kept at the Department of Pathology, Rijeka University School of Medicine. Based on disease recurrence, patients were divided in two groups: patients who developed recurrent disease during the first five post-operative years (N=78) and patients without recurrent disease during a follow-up of minimum 5 years (N=49). The follow-up of patients was subsequently scheduled at control cystoscopy every 3 months for the first two years after TUR, and after that biannually. Recurrence was defined as a written description of a recurrent tumor at any control cystoscopy 6 months after the operation, localized away from the primary tumor bed and the area of the initial resection. By this procedure protocol we wanted to exclude all possible residual tumor masses.

**Figure 1:**
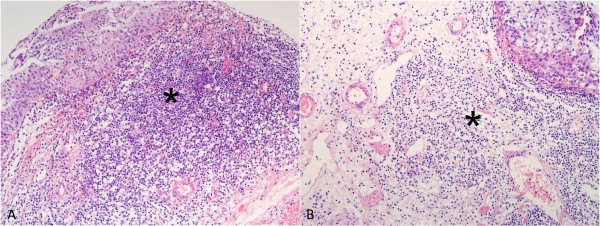
Urothelial tumors (hemalaun-eosin) with strong (A) and moderate (B) mononuclear inflammatory infiltrates in lamina propria (*) predominantly composed of lymphocytes.

Hematoxylin and eosin stained tumor sections were used in order to evaluate the presence of inflammatory cells and to mark the areas with the most pronounced inflammatory infiltrate for construction of tissue microarrays. Positive inflammatory cells were semi quantitatively graded and presence of TILs was categorized as absent (no) or present (yes) as shown in Figure 1.

### Tissue microarray construction (TMA)

Paraffin blocks were available for all 127 cases, and TMAs were constructed by using a manual tissue arrayer (Alphelys, Plaisir, France). Three tissue cores, each 1 mm in diameter, were placed into a recipient paraffin block. Normal liver tissue was used for orientation. Cores were spaced at intervals of 0.5 mm in the x- and y-axes. One section from each TMA block was stained with hematoxylin and eosin to confirm the presence of tumor tissue. Serial sections were cut from TMA blocks for immunohistochemical staining. Three to four μm thick sections were placed on adhesive glass slides (Capillary Gap Microscope Slides, 75 μm, Code S2024, DakoCytomation, Glostrup, Denmark), left to dry in oven at 55°C overnight, deparaffinized and rehydrated.

### Immunohistochemistry and scoring

Tumor samples were processed for immunohistological analysis in a Dako Autostainer Plus (DakoCytomation Colorado Inc, Fort Collins, CO, USA) according to the manufacturer’s protocol using Envision peroxidase procedure (ChemMate TM Envision HRP detection kit K5007, DakoCytomation, Glostrup, Denmark).

The sections were incubated with the primary antibodies, anti-COX-2 mouse monoclonal antibody (clone CX-294, code M3617, DAKO, Denmark), dilution range 1:100. The immune reaction was amplified using the appropriate secondary antibody and the Streptavidin–Biotin–Peroxidase HRP complex (code K5001, DAKO, Denmark). Sections were then developed using 3,3^′^-diaminobenzidine tetrahydrochloride chromogen (DAB, code K5001, DAKO, Denmark), under microscope control. The sections were counterstained with Mayer’s Hematoxylin. Quality control performed by external and internal negative and positive controls was necessary to monitor the accuracy of tissue processing, staining procedures and reagents effectiveness. The primary antibody specificity sought to be assessed by their negative controls.

COX-2 immunohistochemical expression was quantified and scored by assessing a proportion of percentage as an intensity score according to Allred’s scoring protocol [19]. The assigned proportion score represented the estimated proportion of positive-staining cells (0, none; 1, <1/100; 2, 1/100 to 1/10; 3, 1/10 to 1/3; 4, 1/3; to 2/3; and 5, >2/3). The intensity score assigned represented the average intensity of positive cells (0, none; 1, weak; 2, intermediate; and 3, strong). The proportion and intensity scores were combined to obtain a total score, which ranged from 0 to 8. Total score 0–2 was assessed as negative (0), score 3–5 was assessed as weak positive (1) and 6–8 as strong positive (2). All slides were scored by investigator who was blinded to patient clinical information.

For the purpose of further statistical analysis, the mean values of immunohistochemical staining of all three tissue microarrays were used.

### Statistical analysis

Statistical analysis was performed using MedCalc for Windows, version 12.3.0.0 (MedCalc Software, Mariakerke, Belgium). In the first part of the work the classical descriptive methods were used as well as the Chi-square test for the comparison of proportions. Mann–Whitney test was used to compare the medians. The method of logistic regression was used in order to compute the odds ratio for predictors of recurrence in univariate and multivariate manner. The AUC value was used to evaluate the quality of predictive model. The significance level in all tests was 0.05.

## Results

### Clinicopathological characteristics and immunhistochemical findings in NMIBC

Clinicopathological characteristics of 127 patients with solitary, papillary NMIBC unrolled in the present study are summarized in Table 1. All clinical details along with adequate tumor samples, at the time of diagnosis and in follow up period, were available for all patients. None of the patients was lost to follow up. The median age at diagnosis for the patient cohort was 73 years (range 41–87), with a male to female ratio of 3.3:1, and a median follow up period of 37 months (range 6–155). Tumors divided according to their size in ≤3 or >3 cm, according to their pathology in groups of papillary urothelial neoplasms of low malignant potential (PUNLMP) and low grade papillary urothelial carcinoma (LGPUC), and according to their stage in Ta and T1 were nearly equally distributed.

**Table 1:** Clinical features of patients with non-muscle invasive bladder carcinoma along with pathological and immunohistochemical characteristics of the resected tumors

**Clinical, pathological and immunohistochemical features (N=127)**	
**Gender**	**Cases (%)**
Male	94 (74)
Female	33 (26)
**Age (years, median, range)**	73 (41–87)
**Tumor size:**	**Cases (%)**
> 3 cm	59 (46)
≤ 3 cm	68 (54)
**Pathology:**	**Cases (%)**
PUNLMP^†^	54 (47)
LGPUC^‡^	61 (53)
**Disease stage**	**Cases (%)**
**Ta**	54 (46%)
**T1**	64 (54%)
**Median follow-up (months)**	37
**COX-2 Allred score**	**Cases (%)**
0	29 (29)
1	16 (16)
2	54 (55)
TILs*	**Cases (%)**
No	44 (41)
Yes	64 (59)

Note: ^†^PUNLMP indicates papillary urothelial neoplasm of low malignant potential; ^‡^LGPUC, low-grade papillary urothelial carcinoma; *TILs**,** tumor-infiltrating lymphocytes.

**Figure 2:**
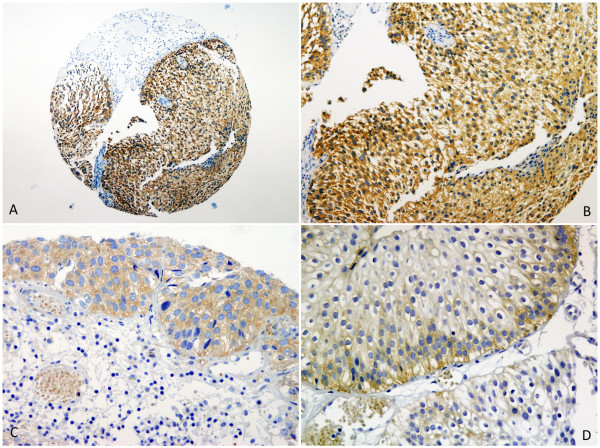
**Immunohistochemical staining for COX-2 on tissue microarrays of urothelial tumors (A, magnification x20).** On higher magnification representative cases demonstrate tumor cells with strong (**B**), moderate (**C**) and weak (**D**) staining (magnification x100).

Immunohistochemical analysis revealed COX-2 expression predominantly granular localized to the cytoplasm of tumor cells. Nuclear staining was not observed. In a few cases immunoreactivity was detected in endothelial cells within the tumor. Low intensity staining was also observed in smooth muscle tissue in some sections. There was noticeable heterogeneity in the intensity and percentage of COX-2 positive cells within the tumor tissues. Positive COX-2 immunoreactivity scored according to Allred was presented in 70 (71%) while 29 (29%) tumors were negative. Among positive tumors 16 (16%) show weak and 54 (55%) strong immunostaining. Representative cases of tumors with moderate and strong COX-2 staining are presented in Figure 2.

Tumor infiltrating lymphocytes as shown in Figure 1 were presented in majority (64; 59%) of cases, while 44 cases (41%) did not reveal mononuclear infiltration in tumoral stroma.

### COX-2 expression and TIL in correlation with recurrence of disease

Main objectives of the present study were to examine possible association between COX-2 expression and TILs with regard to recurrence of disease, as well as their mutual relationship which revealed no significant association (p=0.892).

Clinicopathological parameters included in the above mentioned analysis are presented in Table 2. As it can be seen among the examined parameters pathology and disease stages were different between recurrent and non recurrent disease. In particular, PUNLMP was more presented (61%) among the non-recurrent disease in opposite to LGPUC that was more frequently present (62%) in the recurrent group (p=0.0258, p=0.0258, respectively). According to disease stages there were more patients with recurrence in stage T1 (63%), while those patients without recurrent disease were in stage Ta (61%) (p=0.018).

**Table 2:** Clinicopathologic characteristics, COX-2 expression and TIL in comparison to recurrence of non-muscle invasive bladder carcinoma

	**Recurrent disease**		
**N=127**	**Yes (n=78)**	**No (n=49)**	**P-value**
**Gender**	**Cases (%)**	**Cases (%)**	
Male	58 (74)	36 (73)	0.8834
Female	20 (26)	13 (27)	
**Age (years, median, range)**	71 (41 – 87)	67 (54 – 78)	
**Tumor size:**	**Cases (%)**	**Cases (%)**	
> 3 cm	40 (51)	19 (39)	0.1104
≤ 3 cm	38 (49)	30 (61)	
**Pathology:**	**Cases (%)**	**Cases (%)**	
PUNLMP^†^	26 (38)	28 (61)	0.0258
LGPUC ^‡^	43 (62)	18 (39)	
**Disease stage**	**Cases (%)**	**Cases (%)**	
**Ta**	26 (37)	28 (61)	0,0182
**T1**	46 (63)	18 (39)	
**Median follow-up (months)**	13	75	
**COX-2 score**	**Cases (%)**	**Cases (%)**	
0	27 (45)	2 (5)	0.0001
1	7 (12)	9 (23)	0.2196
2	26 (43)	28 (72)	0.0101
TILs*	**Cases (%)**	**Cases (%)**	
No	32 (53)	12 (26)	0.009
Yes	29 (47)	35 (74)	

Note: ^†^PUNLMP indicates papillary urothelial neoplasm of low malignant potential; ^‡^LGPUC, low-grade papillary urothelial carcinoma; * tumor-infiltrating lymphocytes.

Moreover, statistical analysis demonstrated a difference in tumors COX-2 expression between the analyzed groups, e.g. between recurrent and non-recurrent patients. Possibly, due to small sample size in the group with the score 1 no significant difference was found between recurrent and non-recurrent patients (p=0.2196). In the group with the score 0 and the group with the score 2 there was a significant difference in the proportion of patients with and without recurrence (p=0.0001, p=0.0101, respectively). From these results we can expect that lower score is predictor of recurrence, since there is higher proportion of recurrent patients with score 0 and lower proportion of recurrent patients with score 2.

A significant difference between the recurrent and non-recurrent patients in both groups, with absent and present TILs was also found (p=0.009, p=0.009, respectively). More precisely, higher proportion of recurrent patients in the group with no TILs and lower proportion in the group with TILs were found. Therefore, we can expect the absence of TILs to be the predictor of recurrence.

### Univariate and multivariate analysis of prediction of recurrence

Univariate and multivariate analysis of the influence of COX-2 expression, TILs, tumor size and disease stage on prediction of recurrence (PR) are shown in Table 3.

**Table 3:** Predictors of recurrence: univariate and multivariate analyses

**Predictor**	**Univariate**				**Multivariate**		
	**OR**	**95% CI**	**P-value**	**AUC**	**OR**	**95% CI**	**P-value**
**Tumor size**	1.69	[0.82; 3.49]	0.1541	0.565	1.71	[0.62; 4.69]	0.3007
**Disease stage**	**2.39**	**[1.15; 4.98]**	**0.0194**	**0.607**	**3.29**	**[1.18; 9.19]**	**0.0228**
**TILs**	**0.60**	**[0.40; 0.89]**	**0.0122**	**0.651**	**0.49**	**[0.29; 0.84]**	**0.0092**
**COX-2**	**0.36**	**[0.20; 0.63]**	**0.0004**	**0.691**	**0.27**	**[0.14; 0.53]**	**0.0002**
					p<0.0001, AUC=0.875 (95%CI 0.747; 0.907)		

Note: *OR*= Odds ratio; *CI* = Confidence interval; *AUC* = Area under the curve; *TILs* = tumor-infiltrating lymphocytes.

The odds ratio for each predictor was computed using the method of logistic regression. As we can see the tumor size is not the significant predictor in univariate, as well as is in multivariate manner. Disease stage is in both analysis significant and positive predictor, which means that the odds for a recurrence in cases with stage 1 are 2.39 times higher than in cases with stage 0. In both manners the TILs and COX-2 are negative predictors. The positive change in score for COX-2 (score higher for 1) produces 0.36 times lower risk, or that the odd for not getting a recurrence is 1/0.36=2.77 times higher. The TILs is also significant negative predictor of recurrence with OR 0.31 in univariate manner and 0.23 in multivariate analysis. The AUC value as a classification parameter which gives us the quality of predictive model was used. In our case we have the AUC 0.875 which means that our model has very high discriminating power.

## Discussion

There is large evidence implicating COX-2 activity in the development of UBC. Yet, the results obtained in the present study demonstrated that low COX-2 instead of high COX-2 score could predict the recurrence of NMIBC. These results open many questions related to the methodology used in various studies, as well as the complexity in understanding the role of different biological markers in tumorigenesis.

In the present study we used TMA instead of whole tissue section for immunohistochemical analysis. Gudjonsson et al. in their study analyzed COX-2 among different markers on TMA and they found that proteins assessed had no predictive value for recurrences of Ta bladder cancer (BC) [20]. The concerns have been raised by the authors regarding the methodology and generalization of results obtained with TMA in immunohistochemical analysis. Even so we believe that conclusion was made despite the facts that relatively small number of patients (N=52) was analyzed. TMA construction was done with at least three 0.6 mm punch cores and specimens were heterogeneous, including single and multifocal tumors. In order to minimize these problems the present study was conducted on relatively larger number of the patients (N=127), with only single tumor at the time of diagnosis, and with specimens where at least three 1 mm punch could be selected for TMA construction. Nevertheless, the obtained results indicate that different methodology and also diverse scoring system for protein expression could be the reason for different results and conclusion made in different papers.

Previous studies have been conducted mostly on heterogeneous groups of patients with superficial and invasive UBC. One of the first papers in which the expression of COX-1 and COX-2 were analyzed in human invasive TCC of the urinary bladder samples was by Mohammed and coworkers [21]. COX-2 was not expressed in normal urinary bladder samples but was detected in invasive TCC, noninvasive TCC samples, and in cases of carcinoma in situ. Authors concluded that COX-2 may play a role in BC and support further study of COX-2 inhibitors as potential antitumor agents in human BC. After this study several other ones showed significant increase of COX-2 expression with advancing tumor grade and T stages of the disease [22,23], and not only with disease progression but also with BC specific survival [24].

Opposite to the role of COX-2 in tumor progression there are also studies where COX-2 expression was not associated with primary tumor stage, lymph node status, histological grading, overall and disease-free survival [25]. Wulfing et al. also did not find COX-2 expression associated with TNM staging, histological grading, overall or disease free survival, but a significant relation to the histological subtype (transitional vs. squamous cell carcinoma) was present [26]. Subgroup of chemotherapy patients demonstrated a significant correlation of strong COX-2 expression with worse overall survival time. The authors concluded that further experimental and clinical studies were needed to elucidate if COX-2 inhibition can serve as an additive therapy to chemotherapy of BC.

There are also several studies analyzing COX-2 expression only in NMIBC. In the CIS group, COX-2 expression was significantly associated with disease recurrence and progression, but not with BC related survival (as in our analysis, data not shown), while in the stage T1 the TCC COX-2 expression was not associated with clinical or pathological parameters or clinical outcome [27]. Kim and coworkers in their study selected only T1G3 TCC who had undergone complete TUR [28]. In that case COX-2 expression was statistically significant in predicting both recurrence and disease progression, while patients’ age, shape and multiplicity of tumors were not significantly predictive. Thus patients, according to authors’ conclusion, with COX-2 positive superficial BC may need to be followed up more vigorously. In the paper of Okajima et al. with only 5 and 6 samples of superficial BC cases with and without recurrence, respectively, after TUR, more COX-2 protein samples in the cases with recurrence than in cases without recurrence were found [29]. Even though the number of cases examined is small, as the authors stated, this result supports their hypothesis that COX-2 contributes to superficial BC recurrence, thus selective COX-2 inhibitors can be a candidate chemo preventive agents for reoccurrence.

Our results differ from the above mentioned because loss of COX-2 was a predictive marker of tumor recurrence. We believe this can be explained by very complex, even different role of this enzyme during tumorigenesis. There is no doubt that COX-2 expression is sequentially up-regulated from normal to chronic cystitis and to malignant changes [30]. Also there are evidence that COX-2 is involved in angiogenesis in BC, as described in the paper where COX-2 promoted vessel proliferation in the tumor zone of pTa/pT1 NMIBC [31]. Moreover, COX-2 is probably up-regulated during tumor progression, as mentioned above, but we believe its role in initial stage is very complex. We hypothesize that, in the initial tumor stage, COX-2 is probably associated with host immunological/inflammatory response and, as such, it could be a marker of sufficient anti-tumor response while loss of COX-2 expression may be indication for the selection of patients for additional immunotherapy. This supposition is also supported by our finding that absence of TILs became the predictor of recurrence. In our study we could not find the association between COX-2 and TILs although there is study by Himly and coworkers in which the association between COX-2 expression and T lymphocyte subsets, CD4+ and CD8+, was confirmed [23]. However, our previous work confirmed the proportion of CD4+ cells and Granzyme B+ TILs being significantly higher in non-recurrent group of patients [32].

## Conclusion

Although further studies are necessary to fully elucidate the mechanism involved, the present study showed that higher COX-2 expression and present TILs resulted as a negative predictor of recurrence in NMIBC. On the other hand, patients with lower value of both analyzed parameters should be probably selected for adjuvant therapy.

## Competing interests

The authors declare that they have no competing interests.

## Authors’ contributions

TT participated in study design, conceived the study and participated in coordination. KK added clinical data. SŠ actively performed part of immunohistochemical analysis. EB conceived the study and participated in coordination and performed statistical analysis. ŽF participated in study design and coordination. NJ advised and led the whole group as coordinator. All authors read and approved the final manuscript.
